# Focus clinical ultrasonography: again competency differs from the patient outcome

**DOI:** 10.1186/s13089-022-00258-6

**Published:** 2022-02-09

**Authors:** Luigi Vetrugno, Marco Ventin, Salvatore Maurizio Maggiore

**Affiliations:** 1 Department of Medical, Oral and Biotechnological Sciences, University of Chieti-Pescara, Chieti, Italy; 2 Department of Anesthesiology, Critical Care Medicine and Emergency, SS. Annunziata Hospital, Chieti, Italy; 3 Department of Surgery, Massachusetts General Hospital, Harvard Medical School, 55 Fruit St, Boston, MA 02114 USA; 4 Department of Innovative Technologies in Medicine and Dentistry, Gabriele d’Annunzio University of Chieti-Pescara, Chieti, Italy

**Keywords:** Clinical ultrasound, Education, Internal medicine, Proficiency, Patient outcome

Dear Editor,

We read with great interest the article recently published on JAMA Network Open by Ximena Cid-Serra et al. about the effect of a multiorgan focused clinical ultrasonography (FCU) on length of stay (LOS) in patients admitted to the internal medicine unit with a cardiopulmonary diagnosis [1]. The authors concluded that when multiorgan FCU is added to the initial clinical examination and compared to standard care without FCU, there is no difference in hospital LOS, incidence of readmission within 30 days, and general costs.

The aim of this letter is to comment on whether, based on the conclusion of this study, FCU should be considered a positive, neutral, or harmful tool for this court of internal medicine patients, because the conclusions may become important for readers.

Our first comment is that the treating team of the study performed a standardized FCU assessment with detailed reports without any consequent standardized recommendation on how to modulate the patient’s management. Second, in the study by Ximena Cid-Serra et al. shortness of breath was the most common presenting complaint of the population enrolled, present in 207 patients (83.4%), and our attention was caught by the fact that no one of these patients exhibited cardiac diastolic dysfunction; this result sounds not usual as it is well known that about 50% of patients with congestive heart failure have preserved ejection fraction. The difference between the two diagnoses may have an influence on the outcome. Third, this study used a single FCU examination without any repetition during the time course of the patient’s management, which is beyond the main principles and philosophy of point-of-care ultrasound. In our humble opinion, these three points are main concerns that undermine the conclusion but even the general layout of this study.

In the literature, few studies have shown that any diagnostic tool can improve a patient’s outcome without a treatment protocol or a specific goal to reach; we may cite the example of the goal-directed therapy guided by hemodynamic monitoring tools [2]. Mozzini et al. reported that repeated lung ultrasound examinations impact the patient management by reducing the hospital LOS of 1 day in patients with acute decompensated heart failure (ADHF) admitted to the internal medicine ward [3]. We also know from the literature that longer hospital stays are not related to better clinical outcomes in ADHF [4]. Of note, there is also no evidence that reducing the cost of care will improve the clinical outcomes in patients with ADHF. More likely, the outcome is strongly influenced by the clinical setting, the hemodynamic situation and the intervention done, and partially by the institution, country, and the technological level of the healthcare system where the patients are cared.

Thus, should we draw a negative interpretation of these results and conclude that FCU has no influence in the patient’s management in the clinical setting where these authors operate? Probably this is a wrong conclusion, as in literature many data support different conclusions. Moreover, it is not always useful and even not appropriate to test the effect of diagnostic tools on the clinical outcome. For instance, we do not know any randomized controlled trial showing that transesophageal echocardiography can improve patient’s survival in a cardiac surgery setting or other trials proving that pulse oximetry has an influence on perioperative events [5, 6]. Similarly, tube feeding lacks demonstration of meaningful benefits in patients with advanced dementia [7]. Nevertheless, no one can debate their use. Clinical benefits from many medical devices in specific clinical contexts are not proved by evidence but remain intuitive, and clinicians take many decisions under conditions of uncertainty [8]. In the case of ultrasound, putting competence into practice yields high performance, which does not necessarily imply an advantageous patient outcome.

## Data Availability

Not applicable.
